# Evaluating brief motivational and self-regulatory hand hygiene interventions: a cross-over longitudinal design

**DOI:** 10.1186/s12889-015-1453-7

**Published:** 2015-02-04

**Authors:** Pempa Lhakhang, Sonia Lippke, Nina Knoll, Ralf Schwarzer

**Affiliations:** Health Psychology Division, Freie Universität Berlin, Habelschwerdter Allee 45, 14195 Berlin, Germany; Jacobs University Bremen gGmbH, Campus Ring 1, 28759 Bremen, Germany; Institute for Positive Psychology and Education, Australian Catholic University, 25A Barker Road, Strathfield, NSW 2135 Australia; Department of Psychology, University of Social Sciences and Humanities, ul, Ostrowskiego 30b, 53-238 Wroclaw, Poland

**Keywords:** Motivation, Self-regulation, Handwashing, Intention, Self-efficacy, Planning, Young adults

## Abstract

**Background:**

Frequent handwashing can prevent infections, but non-compliance to hand hygiene is pervasive. Few theory- and evidence-based interventions to improve regular handwashing are available. Therefore, two intervention modules, a motivational and a self-regulatory one, were designed and evaluated.

**Methods:**

In a longitudinal study, 205 young adults, aged 18 to 26 years, were randomized into two intervention groups. The Mot-SelfR group received first a motivational intervention (*Mot*; risk perception and outcome expectancies) followed by a self-regulatory intervention (*SelfR*; perceived self-efficacy and planning) 17 days later. The SelfR-Mot group received the same two intervention modules in the opposite order. Follow-up data were assessed 17 and 34 days after the baseline.

**Results:**

Both intervention sequences led to an increase in handwashing frequency, intention, self-efficacy, and planning. Also, overall gains were found for the self-regulatory module (increased planning and self-efficacy levels) and the motivational module (intention). Within groups, the self-regulatory module appeared to be more effective than the motivational module, independent of sequence.

**Conclusions:**

Self-regulatory interventions can help individuals to exhibit more handwashing. Sequencing may be important as a motivation module (Mot) first helps to set the goal and a self-regulatory module (SelfR) then helps to translate this goal into actual behavior, but further research is needed to evaluate mechanisms.

## Background

More than 100 years of evidence conclusively demonstrates that handwashing reduces the risk of infection [1] and that it is one of the most effective ways of preventing the transmission of infectious agents [2]. However, only 31% of men and 65% of women wash their hands after using a public restroom [3] and handwashing is even less common among young adults [4]. Despite this, non-compliance to hand hygiene is rarely studied in this age group [5]. Particularly in one of the world’s largest youth population, India, with around 66% of the total population under the age of 35 [6], handwashing was hardly systematically studied before. Thus, this was one goal of the current study.

In the hand hygiene literature, previous studies were mainly done among healthcare workers (HCW) [2] and very few of them were generalizable to the population of adults, much less to youth. Moreover, these studies were lacking quantitative research methods, and very few could be found testing strategies to enhance handwashing empirically [4]. In one of the few studies addressing a handwashing campaign, education was addressed [7], but it has been shown to be ineffective in handwashing promotion. Authors suggested testing more effective interventions and their differential impact in changing behavior [8] especially in a student residence hall environment and university campus [9,5,10]. Thus, the current study aimed at focusing on university students and promoting handwashing in this sample with theory-based interventions, because interventions are imperative for successful health behavior promotion [11].

### Motivational and self-regulatory strategies for health behavior change

The World Health Organization prioritized hand hygiene as an intervention of universal relevance across developed and developing countries in 2005 [12]. Interventions should include the general principles guiding the development of health promotion programs and employed strategies based on relevant evidence coupled with an understanding of the underlying mechanisms [13,14]. Such evidence-based strategies to enable health behavior change include addressing self-efficacy and self-regulatory skills such as planning [15], as suggested by the health action process approach (HAPA) [16].

According to the HAPA, individuals first need to become motivated to adopt health behaviors, i.e. form an intention. This can be supported by strategies enhancing psychological variables such as *risk perception*, *outcome expectancies,* and *self-efficacy*. The HAPA assumes that after forming a behavioral intention, i.e., as soon as individuals are motivated, they need self-regulatory skills such as planning and further self-efficacy to translate their intentions into actual health behavior and to maintain the behavior over time [16]. A study on fruit and vegetable intake among young adults highlighted the advantage of a self-regulatory intervention over a motivational intervention [17]. This also needs to be investigated in the domain of hand hygiene.

### Motivational factors: risk perception and outcome expectancies

According to *HAPA, risk perception* can be a starting point for contemplating health behavior change although it is not regarded as a powerful predictor of most behaviors. *Outcome expectancies* are the perceived consequences of adopting the health behavior, which are supposed to lose their predictive power after a personal decision has been made. To form a behavioral intention, one also needs perceived self-efficacy which is the belief in one’s capability of performing a desired action [18].

### Self-regulatory factors: perceived self-efficacy and planning

Perceived self-efficacy is the confidence in one’s ability to execute a difficult or resource-demanding behavior and the capability of performing the actions that are required to attain a desired end state. Moreover, self-efficacious individuals invest more effort into achieving their goals especially in the face of barriers [19]. The barrier here is not the technical difficulty of hand hygiene, but rather the regular performance of this activity as an integral part of daily life. Self-efficacy predicts a range of health behaviors including hand hygiene [20]. Self-efficacy is not only imperative for setting the goal to change behavior (motivational effect) but also for translating the goal into behavior and maintaining the behavior after the first enactment (volitional effect).

Intention to change a behavior is central to most health behavior promotion programs [21] but intentions can be unstable, and as a consequence people might not actually take up an action, for example, when they lack control over their behavior [22]. The “black-box” nature of the underlying psychological processes that lead from intention to action has been labelled intention-behavior-gap [23]. To bridge this intention-behavior-gap, it has been shown that forming specific *action plans* of when, where, and how to act can increase the likelihood of successful implementation of one’s intention. Two types of planning have been introduced: *action planning* (when, where and how to act) and *coping planning* (to identify barriers and strategies to cope with them) [24]. Much research has documented the pivotal role of planning interventions for the uptake and maintenance of a variety of health behaviors [25,26]. However, its effects on a self-regulatory handwashing intervention in comparison to a motivational intervention, has not been tested before.

### Aims and hypotheses

The current study explored whether it would make a difference in which order a set of two brief theory-based psychological intervention arms (motivational and self-regulatory interventions) are presented to improve hand hygiene in young adults. It evaluated a unique research design by comparing these two intervention modules. A crossover design was employed: one group of participants received the motivational intervention (Mot) first, followed by the self-regulatory module (SelfR). The other group received the two modules in the opposite sequence (SelfR-Mot). At the point in time when the order was switched (after 17 days), a second assessment took place allowing to gauge the changes during the first phase. In addition, after another 17 days the third assessment was scheduled to allow for evaluating the final outcomes.

The purpose of this cross-over design was to examine whether it made a difference in which order treatments were presented. According to the theoretical assumptions derived from the HAPA, participants should first become motivated before they acquire self-regulatory skills. Thus, the aim is to determine the optimal sequence for motivational and self-regulatory modules, and it is expected that the motivation-self-regulation (MotSelfR) sequence would be superior in adopting the handwashing goal as well as planning this behavior, compared to the opposite sequence (Hypothesis 1: Mot-SelfR > SelfR-Mot).

However, it may be that some participants do not benefit from such a sequence because they feel patronized by the motivational messages or they perceive them as redundant, as they are already beyond that stage. In such cases, a mere self-regulatory intervention without a motivational precursor would be the better and more parsimonious option.

Moreover, the intervention benefits should not only be documented by changes in handwashing but also by changes in mindsets as reflected by higher levels of self-efficacy and planning. This leads to the following hypothesis: participants who received the self-regulation intervention module would gain more in terms of the volitional outcomes (behavior and planning), whereas those receiving the motivational intervention module would show increases in the motivational outcome (intention), no matter at which point in time (Hypothesis 2).

## Method

### Participants

University students (*n =* 206, including 107 women, mean age = 20.71 years, *SD =* 1.59 with a range from 18 to 26 years) were observed from March 2013 to April 2013 with three assessment points in time over a time span of 34 days. Participants were recruited from a university student residence in New Delhi, India, via a notice by the student council board. Attending the program was voluntary and written informed consent was obtained by the participants. The intervention followed ethical principles regarding research with human participants. The ethics committee which granted approval was the Tibetan Youth Hostel Board of Council, New Delhi, India.

### Research design and procedure

By cluster randomization, participants (*N* = 225) were allocated to two intervention groups using a cross-over design. Intervention sequences were implemented after the baseline measurement. Intervention sequence group 1 (*Mot-SelfR*) received a written *motivational* module after the baseline measurement (Time 1; T1) and a written *self-regulatory* module 17 days later after the post-test (Time 2; T2). The intervention sequence group 2 (*SelfR-Mot*) was treated with a *self-regulatory* module after the baseline measurement, followed by a *motivational* module after T2.

The interventionist resided with the participants during the whole study period and observed students practicing and engaging in the intervention modules. Soap was provided in all wash basins including rest rooms and kitchen, and signs with standard educational messages were placed in rest rooms. Each intervention session lasted 20 minutes, and the measurement intervals were 17 days from T1 to T2, and another 17 days from T2 to Time 3 (T3). The study was conducted by the first author together with four student research assistants, who were blinded completely and, therefore, were not aware of the aims, intervention content, and any other information that could bias the results.

### Measures

*Handwashing* frequency was assessed with three items adapted from a previous handwashing study [27], which examined the frequency of handwashing per day, with plain water, handwashing with soap and water, and frequency of using disinfectant. The item stem, ‘During the last week, I have…’ was followed by the items ‘washed my hands with plain water’, ‘washed my hands with soap and water’, and ‘disinfected my hands with disinfectant’. The responses ranged from 1: 0–5 times; 2: 6–10 times; 3: 11–20 times; 4: more than 20 times.

*Behavioral intention* was assessed with two items (Spearmen’s ρ_T1_ = .52, ρ_T2_ = .65, ρ_T3_ = .75); the item stem ‘I intend to wash my hands properly either with soap or with an alcohol-based solution…’ was followed by the items ‘more than ten times a day’, ‘at least ten times a day’. Responses for intention as well as for self-efficacy and planning were assessed using 4-point scales ranging from 1 = *not at all true* to 4 = *exactly true.* Measures of this type were validated in previous studies [16].

*Self-efficacy* was assessed with six items (α_T1_ = .72, omega = .72, [.60, .80], α_T2_ = .70, omega = .68, [.56, .76], α_T3_ = .75, omega = .74, [.65, .80]), such as ‘I am confident that I can start washing my hands immediately on a regular basis even if others do not wash their hands’ and ‘I am confident that I can frequently wash my hands on a long-term basis, even when it takes a long time to make this a part of my daily routine’.

*Planning* was assessed with six items (α_T1_ = .73, omega = .74 [.64, .80], α_T2_ = .89, omega = .89 [.86, .92], α_T3_ = .81, omega = .82, [.76, .86]), three items measuring action planning (e.g., ‘I have made a concrete and detailed plan regarding when and where to wash my hands (at which occasion)’ and three items measuring coping planning (e.g., ‘To keep my hand hygiene habit in difficult situations…’, ‘…I have made a concrete plan regarding what to do if something interferes with my goal of handwashing’).

Responses were rated on a four-point Likert-type scale ranging from (*1*) not at all true to (*4*) exactly true. Items on planning and self-efficacy were adapted from [16].

#### Intervention content: motivational and self-regulatory modules

Soaps and soap solutions were provided in every toilet and washing areas of the residences during the study period for participants’ use. This was done because providing accessible resources is an obvious necessary component of any hand hygiene intervention [5,28].

Intervention content is described in terms of the *Behavior Change Techniques* [13] (BCT). In the *motivational module (Mot)*, participants received a module with detailed instructions on why and how to wash hands (BCT 4.1), information addressing risk perception (BCT 5.1) and positive outcome expectancies (BCT 1.3) as well as prompts towards intention formation (BCT 7.1). After providing general information about the behavioral risk, participants were instructed to anticipate risks of not washing their hands properly and were encouraged to write down benefits of washing hands (positive outcome expectancies).

In the *self-regulatory module (SelfR)*, the intervention was focused on self-efficacy (BCT 15), and planning (BCT 1.2 and 1.4). After general instruction, participants were encouraged to generate three action plans specifying the timing, frequency, and technique to wash their hands, yielding a total of nine cells (3 plans x 3 details) to fill out (‘have you made a plan on washing your hands to be free of germs? If so, please indicate here your most important plans regarding… *how often* to wash hands, …when to wash, …*how to* wash hands’) and three coping plans, which included both barrier identification and problem-solving (‘If I face difficult situations that might prevent me from washing my hands…, then I plan to overcome them by…’) with a six-cell design (3 × 2: three situations with critical events, each of them with two coping strategies). After each of the three situations (action and coping plans), an item (‘How certain are you that you can follow these plans?’) instructed participants to rate their perceived ability to follow through with the plan on a 4-point scale. These items were designed to boost self-efficacy. To compare their performance with goals and to increase mastery experience (BCT 2.3), participants were prompted to review and visualize their past successes (‘which success experiences had you in washing your hands regularly? Please write here’). The cross-over designed study provided to all participants both types of interventions, the motivational as well as the self-regulatory intervention, either in the theory-based or in the reversed sequence (Mot-SelfR versus SelfR-Mot).

### Analytical procedure

Analyses were conducted with SPSS 22. First, drop-out analyses compared retained participants with those lost after T1 and T2 using *t*-tests for continuous measures and *χ*^2^**-**tests for categorical measures. Second, repeated measures analyses of variance (ANOVA, type III tests of fixed effects) were conducted with the two different interventions (Mot-SelfR versus SelfR-Mot) as a between-subjects factor, whereas handwashing frequency served as a dependent variable. Intention, self-efficacy, and planning served as intermediate outcomes.

All variables were measured at three points in time. Moreover, ANCOVAs were computed with the different groups as a between-subjects factor and the respective outcomes of handwashing frequency, intention, self-efficacy, and planning at T2 and T3 as dependent variables with their corresponding T1 measures as covariates. Assumptions on the properties of the data were tested beforehand such as Levine’s test of equality of error variances and Mauchley’s test of sphericity.

## Results

### Attrition analyses and baseline comparisons

**Figure 1:**
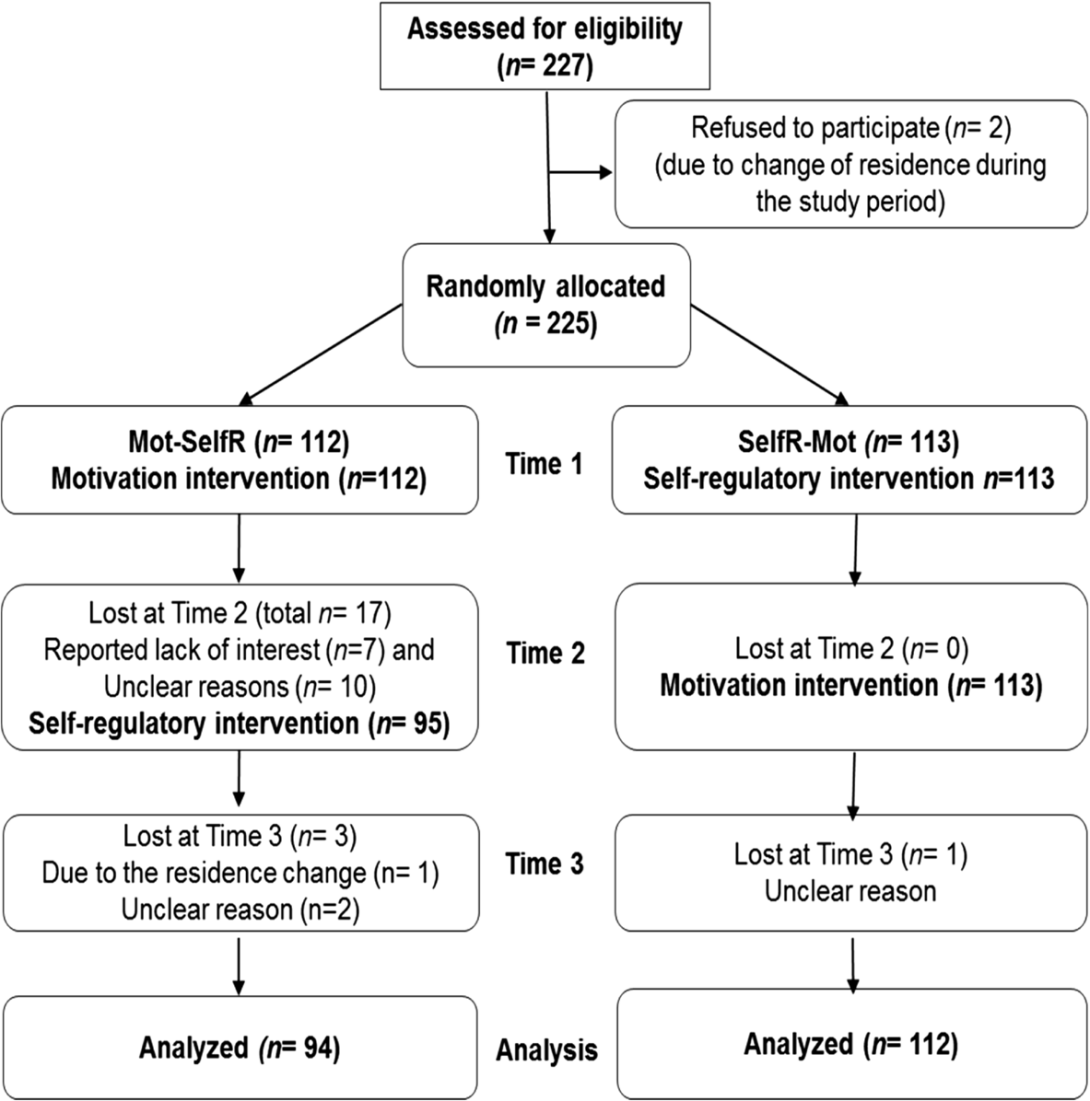
**CONSORT flow chart of the study participants.**

Results at T1 indicated no significant difference between the retained participants and those who discontinued the study after T2 (*n =* 19) regarding the central variables under the study as well as socio-demographic variables (all *p >* .05). Dropouts were excluded from the longitudinal data analyses (see Figure 1).

Missing values were less than 0.5% for handwashing frequency, and less than 1.5% for intention, self-efficacy, and planning at all points in time. Moreover, results revealed no baseline difference (see Table 1) between the two experimental conditions regarding handwashing frequency, intention, self-efficacy, planning, and gender (all *p >* .05). Age differences occurred (*p <* .05), with more of the older study participants assigned to the group SelfR-Mot (*M =* 21.45, *SD* = 1.42) than to the Mot-SelfR group (*M =* 19.83, *SD =* 1.28).

**Table 1:** **Pairwise comparisons with means (** ***SD; range*** **) between the two intervention groups at the different measurement points**

		**Mot-SelfR (n = 94)**	**SelfR-Mot (n = 112)**	
**Outcomes**	**Time points**			***p***
Handwashing	Time 1	6.22 (1.99;3–11)	6.21 (1.56;3–11)	>.05
-	Time 2	7.25 (1.91;3–16)	8.68 (1.74;4–13)	<.001
-	Time 3	9.30 (2.36;3–14)	9.06 (2.72;3–15)	>.05
Intention	Time 1	2.02 (0.86;1–4)	1.95 (0.67;1–3.50)	>.05
-	Time 2	2.86 (0.66;1–4)	2.65 (0.84;1–4)	=.06
-	Time 3	2.59 (0.84;1–4)	3.18 (0.63;1–4)	<.001
Self-efficacy	Time 1	2.24 (0.65;1–4)	2.22 (0.37;1–3.33)	>.05
-	Time 2	2.59 (0.42;1–4)	3.02 (0.58;1.33-4)	<.001
-	Time 3	3.07 (0.59;1–4)	3.16 (0.53;1.67-4)	>.05
Planning	Time 1	1.92 (0.62;1–3.33)	1.84 (0.31;1–2.67)	>.05
-	Time 2	1.83 (0.48;1–3.50)	2.79 (0.62;1–4)	<.001
-	Time 3	2.94 (0.59;1–4)	2.75 (0.63;1–3.83)	<.05

*Note*: T2 took place 17 days after T1, and T3 34 days after T1. There were no baseline differences at T1 (*p >* .05) between the two intervention groups.

### Intervention effects

Prior to the intervention, 96% of the participants did not reach the recommended frequency of handwashing (10 times/day). Moreover, means, standard deviations, and ranges for the two groups’ comparison statistics for all variables are summarized in Table 1.

### Intervention effects on handwashing frequency

**Figure 2:**
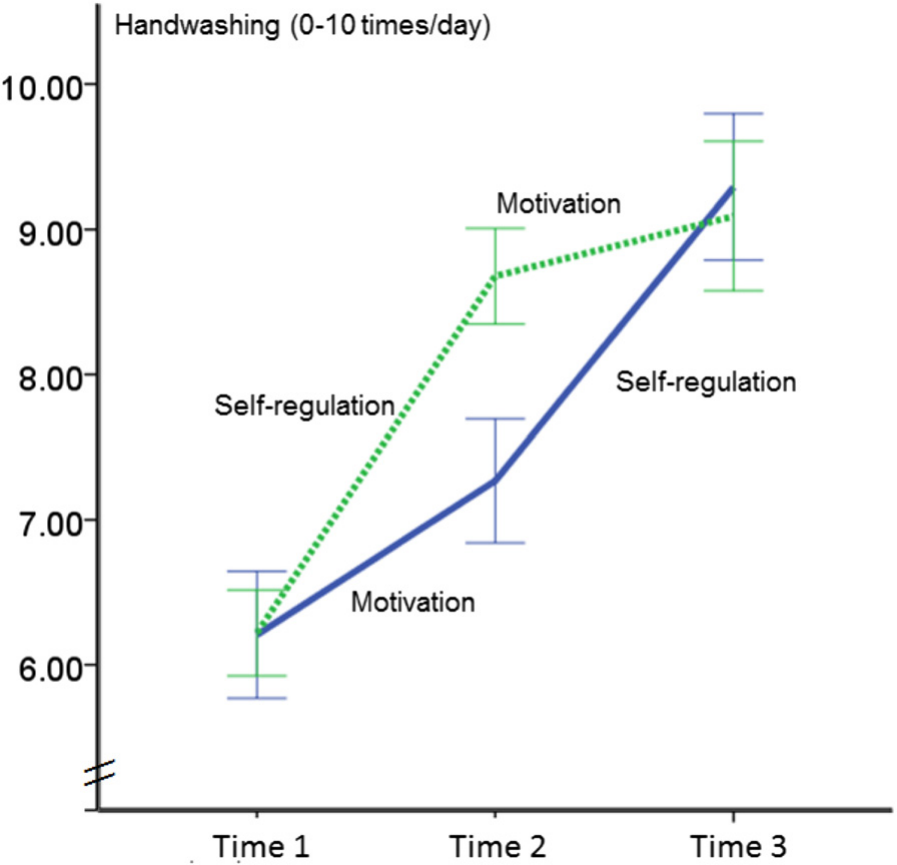
**Handwashing frequency levels for two experimental conditions at three measurement points in time (Mot-SelfR: first motivation, then self-regulation; SelfR-Mot: first self-regulation, then motivation).**

For handwashing, no significant overall difference (*M*_1_ = 7.59, *M*_2_ = 7.99) was observed between the treatment groups, *F*(1, 189) = 3.69, *p* = .06, η^2^ = .02. An effect of time emerged, *F*(2, 378) = 128.12, *p <* .001, η^2^ = .40, and also an interaction between treatment and time was found, *F*(2, 378) = 10.94, *p <* .001, η^2^ = .06. Figure 2 illustrates that after the first intervention module was completed, the SelfR-Mot (self-regulation intervention first) was superior to Mot-SelfR (motivation intervention given first). However, at T3, i.e., after also the second part of the intervention was provided, Mot-SelfR (motivation followed by self-regulation) appeared slightly superior to SelfR-Mot (self-regulation followed by motivation). To test pairwise comparisons, ANCOVAs were computed to test whether the differences between the groups were statistically significant.

The pairwise comparisons revealed that participants having obtained the SelfR intervention at T2 reported a higher mean of behavior in comparison to persons who had obtained the Mot intervention at T2 (*F*(1, 191) = 29.82, *p* < .001, η^2^ = .14). This validates the findings above. However, at T3, after both groups had received all intervention modules (only in opposite order) the difference between the Mot-SelfR group and the SelfR-Mot group was no longer significant *F*(1, 197) = 0.71, *p* = .40.

### Changes in intention, self-efficacy and planning

For *intention*, there was a substantial effect of time, *F*(2, 404) = 95.73, *p* < .001, η^2^ = .32, and a significant interaction between the treatment and time *F*(2, 404) = 19.02, *p* < .001, η^2^ = .09. However, there was no significant specific treatment effect, *F*(1, 202) = 2.29, *p =* .13, η^2^ = .01. The ANCOVA tests at T2 depicted that the Mot intervention resulted in a slightly higher level of intention than the SelfR intervention with *F*(1, 201) = 3.37, *p* < .06, η^2^ = .02. However, at T3, when SelfR-Mot had received the Mot module, they had developed significantly higher intention levels with *F*(1, 203) = 34.29, *p* < .001, η^2^ = .14 in comparison to the Mot-SelfR group after receiving the SelfR module.

**Figure 3:**
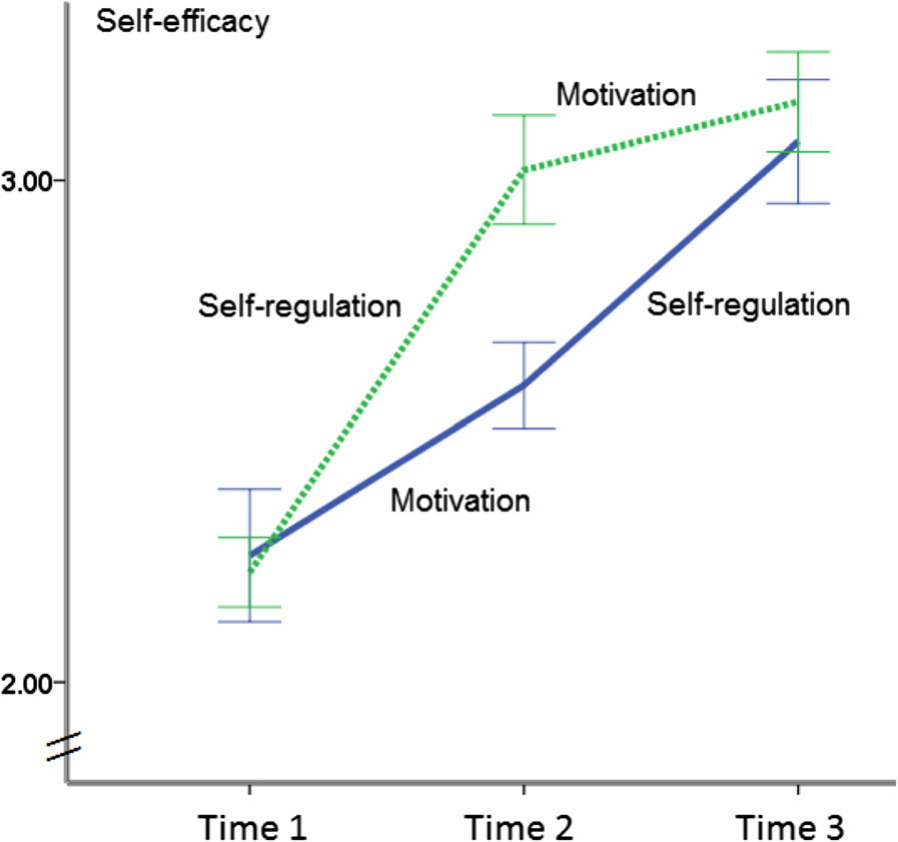
**Self-efficacy levels for two experimental conditions at three measurement points in time (Mot-SelfR: first motivation, then self-regulation; SelfR-Mot: first self-regulation, then motivation).**

For *self-efficacy*, a main effect of time emerged, *F*(2, 404) = 184.32, *p* < .001, η^2^ = .48, as well as a significant treatment effect resulted, *F*(1, 202) = 9.57, *p* < .01, η^2^ = .05. There was also a significant interaction between treatment and time, *F*(2, 404) = 13.45, *p* < .001, η^2^ = .06. Figure 3 displays the patterns of differences in self-efficacy. In pairwise comparisons, at T2, the SelfR module had resulted in a higher level of self-efficacy than the Mot module with *F*(1, 201) = 39.17, *p <* .001, η^2^ = .16. After both groups had received both intervention modules, this difference was maintained descriptively at T3; however, the ANCOVA yielded *F*(1, 203) = 1.44, *p* = .23, η^2^ = .01 and the result indicated that the difference was no longer significant.

**Figure 4:**
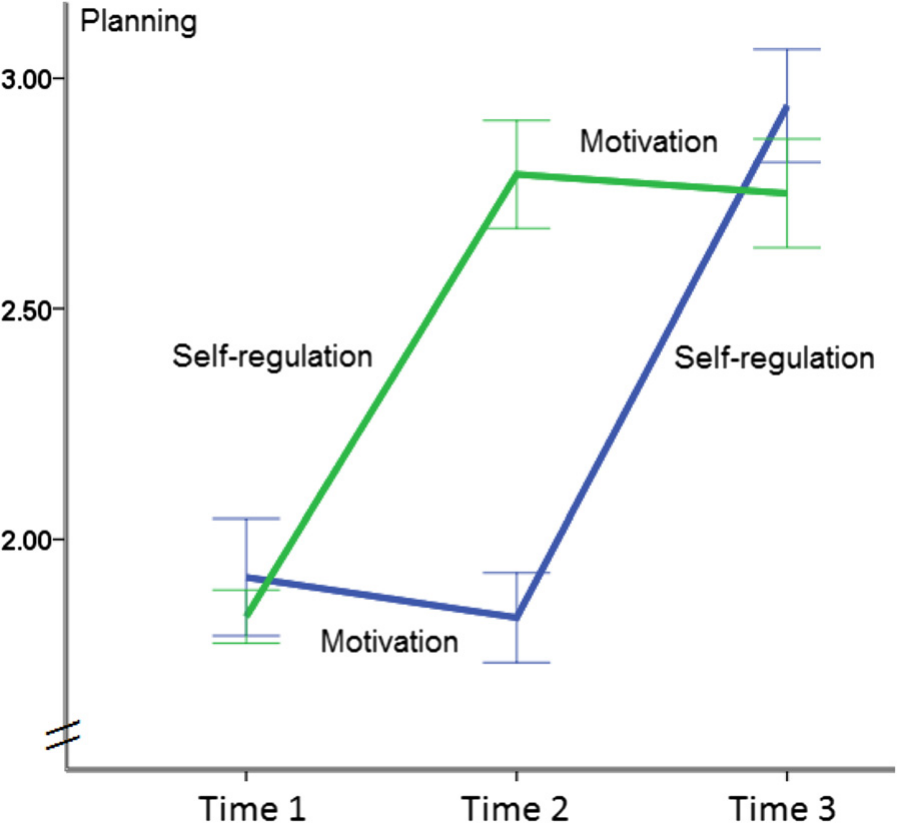
**Planning levels for two experimental conditions at three measurement points in time (Mot-SelfR: first motivation, then self-regulation; SelfR-Mot: first self-regulation, then motivation).**

For *planning*, a main effect of time was found, *F*(2, 404) = 199.59, *p* < .001, η^2^ = .50, along with a significant treatment effect, *F*(1, 202) = 18.24, *p* < .001, η^2^ = .08. There was also an interaction of treatment and time, *F*(2, 404) = 85.70, *p* < .001, η^2^ = .30 (see Figure 4). Pairwise comparisons revealed that at T2, the SelfR module had resulted in a higher level of planning than the Mot module with *F*(1, 201) = 159.21, *p* < .001, η^2^ = .44. However, at T3, when both groups had received both intervention modules, the Mot-SelfR group developed significantly higher planning levels with *F*(1, 203) = 4.16, *p <* .05, η^2^ = .02, than the SelfR-Mot group.

## Discussion

This study explored whether it would make a difference in which order a set of motivational and self-regulatory intervention modules were presented to improve hand hygiene in young adults. The two intervention modules (a motivational intervention and a self-regulatory intervention) were theory-guided, based on the HAPA [16]. Therefore, the intervention modules included psychological constructs such as risk-perception and outcome expectancies (Mot intervention) as well as perceived self-efficacy and planning (SelfR module). Two experimental groups were treated with both interventions, but in two different sequences (Mot-SelfR versus SelfR-Mot), testing their differential effects.

The Mot-SelfR proved to be slightly more effective than the SelfR-Mot to promote handwashing, however, not being significant. This result supports descriptively the first hypothesis and replicates the findings of a previous study on fruit and vegetable intake [17].

Moreover, results yielded that the main differences were observed at T2, when both groups were exposed only to a single intervention, either the motivational or the self-regulatory one. This substantial group difference vanished at T3, after all participants had been exposed to both kinds of interventions. Thus, this research demonstrates that a motivational intervention in itself leads to a mere increase in intention but does not lead to behavior change replicating the previous study [29,30]. The more successful approach lies in the acquisition of self-regulatory skills and the development of confidence in one’s agency confirming the second hypothesis: It was assumed that all study participants receiving the motivational intervention module would show increases in intention, no matter at which point in time. This assumption was corroborated by the data as intention was higher in both groups right after they had received the motivational intervention.

Hypothesis 2 also assumed that planning and actual change in the behavior would be higher after individuals of both groups had received the self-regulatory intervention module. This especially was supported for planning. Regarding the actual behavior change, the assumption can also be supported as not only the mean level of behavior at the measurement points should be evaluated, but also the change in behavior and other test variables. Overall, the motivational intervention, which was mainly educational, did not lead to strong effects in behavior change [31]. It is important that people first set a goal which then needs to be translated into behavior. If individuals have to plan a non-intended behavior, this might have adverse effects. Although it has been frequently shown [32] that conditional planning (if-then) structures such as coping planning are more effective than simply specifying the when, where, and how (action planning), the current study yielded similar effects of action and coping planning on handwashing behavior. This may be due to the habitual nature of the behavior that is produced by particular cues, often as a part of routine and, thus, these two planning components were incorporated here into one single planning construct.

While the results of the current study support the hypotheses, in contrast to previous studies among university students [7,10], no gender differences in the handwashing compliance rate were found (*p >* .05). Thus, the interventions were equally effective in men and women.

There are some limitations. Assessments were self-reported and handwashing frequency was measured retrospectively. For more objective assessments, one could use video cameras and Smart Soap (containing devices that record usage) [33]. To examine more potential mechanisms of the two interventions and, thus, account for the complexity of a behavior-change process, effects on other constructs such as action control (i.e., monitoring one’s progress, comparing performance with goals, and investing more effort if needed) should also be tested. To address the stage of change, future work might also consider a segmentation of the audience in terms of the participants’ levels of previous behavior and concurrent motivation [16]. Further, it could be tested whether matched interventions work better than the mismatched treatments. However, as in this study the baseline handwashing compliance rate was only 4%, it was assumed that almost all participants were initially unmotivated to adopt handwashing 10 times daily. Therefore, no distinction was made between participants in terms of intenders, non-intenders, and actors. Rather, the effectiveness of two intervention modules (Motivation and Self-regulation) was evaluated and a large proportion of participants seemed to have benefitted from both interventions, independent of their stages of change.

It remains unclear whether the low initial compliance rate was due to lack of awareness, lack of motivation, or lack of self-regulatory skills. We assume that in educated college students, a moderate level of hygiene awareness and motivation is given, which then would mean that the translation of intention is at stake. Under such circumstances, a brief self-regulatory treatment, as provided in this study, might be a useful shortcut towards the target behavior, without the need to provide lengthy educational messages.

In the analyses, we did not address possible mechanisms of behavior change that involve intentions, planning, and self-efficacy because this is beyond the present scope and constitutes a different research question. For example, various studies have examined the moderating role of self-efficacy in the volition phase [34]. An interaction between planning and self-efficacy on physical activity has been found [35] as well as an interaction between intention and self-efficacy on planning [36].

## Conclusions

The findings lead to implications for developing health behavior change interventions in public health settings. In promoting hand hygiene among young adults, the implementation of brief self-regulatory skill training appears to be promising, whereas an educational program that provides information to build motivation seems to be less effective. It does make a difference in which order the two intervention modules are presented. The sequence in which individuals were motivated first and then guided to develop their self-regulatory skills appears to be more intuitive and in line with major theories [16] than the opposite sequence, but, on the other hand, behavior change was achieved with the self-regulatory module alone, questioning the usefulness of a motivational prelude to this module.

Overall, this research has explored in a unique way the sequencing of different health behavior intervention modules and elucidated the proximal predictors of changing hand hygiene behaviors, in this case handwashing, and, thus, contributes to the emerging literature on the developments of health behavior change techniques.
